# Genetic diversity within *Miscanthus × giganteus*: Evidence from morphological traits and ITS rDNA in European accessions

**DOI:** 10.1007/s11033-026-12301-z

**Published:** 2026-07-15

**Authors:** Hana Auer Malinská, Diana Polanská Nebeská, Michaela Kocholatá, Anna Erol, Josef Trögl

**Affiliations:** 1 Department of Biology, Faculty of Science, Jan Evangelista Purkyne University in Usti nad Labem, Pasteurova 3632/15, Usti nad Labem, 40096 Czech Republic; 2 Centre for Nanomaterials and Biotechnology, Faculty of Science, Jan Evangelista Purkyne University in Usti nad Labem, Pasteurova 3632/15, Usti nad Labem, 40096 Czech Republic; 3 Clinical Research Centre, Medical University of Bialystok, ul. Jana Kilińskiego 1, Bialystok, 15-089 Poland; 4 Department of Environmental Chemistry and Technology, Faculty of Environment, Jan Evangelista Purkyne University in Usti nad Labem, Pasteurova 3632/15, Usti nad Labem, 40096 Czech Republic

**Keywords:** biomass, genetic variability, ITS (internal transcribed spacer), *Miscanthus × giganteus*, rDNA

## Abstract

**Background:**

*Miscanthus × giganteus* (M × g) is a sterile allotriploid hybrid (*M. sacchariflorus × M. sinensis*) widely used for biomass production and generally considered genetically uniform due to clonal propagation and a strong founder effect. However, phenotypic variation observed in European-grown material suggests that this assumption may be oversimplified.

**Methods and Results:**

This study compared biometric, morphological, and genetic characteristics of four M × g accessions used for agronomy field trials in Croatia (HR), Ukraine (UA), Germany (DE), and the Czech Republic (CZ). Biometric analysis revealed that the HR accession produced 1.6–2-fold higher biomass yield, primarily associated with a 1.5–3-fold increase in sprout number. The HR accession also exhibited distinct morphological traits, including stem pigmentation and trichome presence. Sequencing of nuclear ribosomal DNA internal transcribed spacer markers (ITS1 and ITS2) identified seven polymorphic sites (four in ITS1 and three in ITS2) three of them unique to the HR accession, whereas UA, DE, and CZ displayed identical sequences. *In silico* comparison with reference sequences confirmed and further highlighted the marked divergence of the HR accession. Notably, variation at position 195 bp in ITS2 indicated fixation of a single ribotype in HR compared to heterozygosity in the remaining accessions.

**Conclusions:**

The results suggest the presence of limited genetic variation within M × g, despite its predominantly clonal propagation. The observed association between ITS polymorphisms and phenotypic traits in the HR accession indicates that intra-specific variation may contribute to differences in biomass performance. These findings highlight the potential for identifying and utilizing divergent accessions in future breeding and selection programs, while also suggesting that the assumption of strict genetic uniformity in M × g may require reconsideration.

**Graphical abstract:**

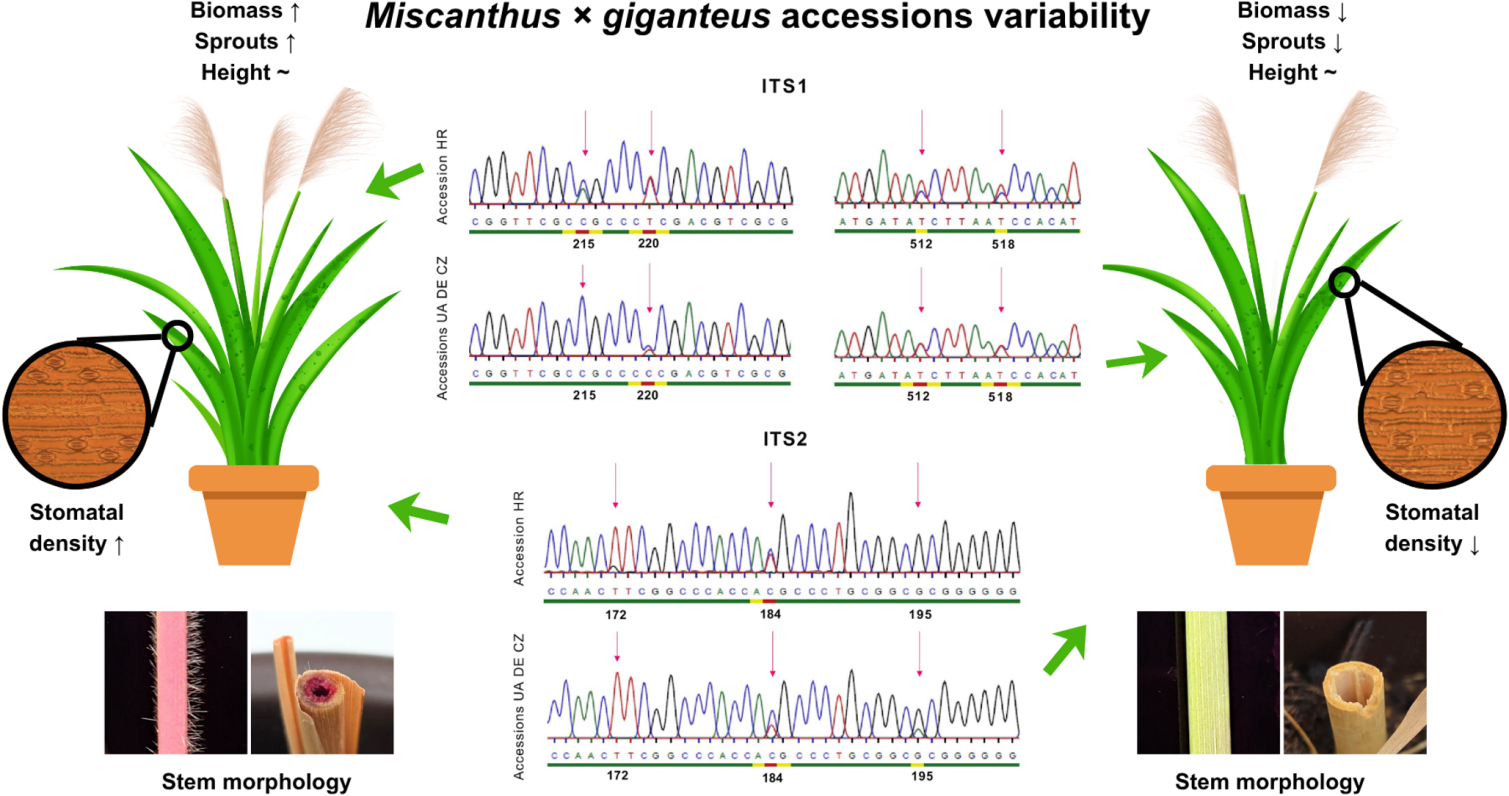

**Supplementary Information:**

The online version contains supplementary material available at 10.1007/s11033-026-12301-z.

## Introduction

The genus *Miscanthus* comprises perennial C4 grasses native to East Asia. Among these, the sterile hybrid *Miscanthus × giganteus* (M × g), an allotriploid (2n = 3x = 57) arising from a cross between diploid *M. sinensis* and tetraploid *M. sacchariflorus*, has attracted considerable attention as a biomass crop. First collected in Yokohama, Japan in 1935 and introduced to Europe as an ornamental plant, M × g was systematically evaluated for energy production only from the 1980s onward [1]. The crop combines high productivity and low input requirements: it can remain productive for 15 to 20 years. It has low nutritional demands and is able to grow on low quality soils [2]. In recent years, M × g biomass has been explored for use of low-cost bioethanol, biogas, and biomaterial production [3, 4]. Despite these advantages, widespread cultivation remains limited, largely due to genetic constraints inherent to this sterile hybrid. The parents of M × g, as well as the hybrid itself, originate from Southeast Asia, a climate markedly different from temperate Europe or North America. When grown in these regions, rhizomes frequently suffer frost damage at temperatures below − 3.5 °C, with complete mortality occurring around − 6 °C [5]. The plant is most vulnerable during the first one to two years after establishment, before the root system is fully developed. Studies of *M. sinensis* genotypes originating in Japan or Siberia show that these cultivars display superior cold tolerance, prompting efforts to identify resistance genes within these populations [6, 7].

The central obstacle to increasing M × g resilience lies in its genetic uniformity. While genetic diversity in the parental species (*M. sinensis* and *M. sacchariflorus*) has been well described [8, 9], cultivars of M × g grown worldwide remain remarkably homogeneous. This uniformity is mainly driven by two factors: the sterility of the hybrid, which restricts reproduction to clonal propagation via rhizome division, and a pronounced founder effect [1, 10, 11]. Mostly used cultivars of M × g are “Illinois” and “Axel Olsen” which were brought to Europe in 1935 [11–15]. The consequences of genetic bottleneck are significant. Estimates based on genetic analyses suggest that the genetic relatedness among M × g individuals approaches 0.94 [16]. This high level of homogeneity leaves the crop vulnerable to pathogens or pests and moreover, complicates genetic breeding efforts [17]. In this case, breeding for desired traits like cold tolerance, increased biomass yield or biomass quality is extremely complicated. Genes associated with these traits have not been identified in M × g, which is likely a consequence of its extremely limited genetic diversity. The only possibility is search for variability in other cultivars or natural parental species [8, 9, 16, 18]. Unfortunately, the situation here is considerably more complex [10, 19–22]. Hodkinson et al. [11] demonstrated through AFLP analysis that many accessions labelled *M. sacchariflorus* in germplasm collections were in fact misidentified M × g clones. Furthermore, *M. sacchariflorus* itself may have hybrid origins, with one genome derived from *M. sinensis* and another from an unidentified species [23]. The phylogenetic relationships within the genus remain questioned, complicating efforts to reconstruct the parentage of existing hybrids or create new ones with predictable characteristics. New methods like detailed genetic studies [10, 24] and high-throughput sequencing [25] have begun to clarify this complexity. Mitros et al. [25] provided a chromosome-scale assembly of the *Miscanthus* genome, revealing its paleopolyploid history and providing a clearer framework for understanding hybrid origins. Furthermore, recent efforts to overcome the ‘Illinois’ genetic bottleneck have shifted focus toward developing new seed-based hybrids [26, 27]. These novel crosses aim to combine the high yield of *M. sacchariflorus* with the cold hardiness of *M. sinensis*, or different parental species giving possibility for broader environmental adaptation. The modern breeding research is increasingly turning towards genome editing technologies. Recent advancements in CRISPR/Cas9-mediated gene editing have opened new avenues for the precision improvement of agronomic traits also in *Miscanthus* [28].

Despite the widely held assumption of genetic uniformity in M × g, an important knowledge gap remains regarding the extent of intra-specific variation among accessions that have been independently propagated and maintained across Europe. In particular, it is still unclear whether phenotypic differences observed under field conditions are driven by underlying genetic polymorphisms or whether they primarily reflect environmental effects. Moreover, previous studies have primarily focused on interspecific diversity within the genus *Miscanthus*, whereas intraspecific variation within M × g has received comparatively less attention [11, 17], especially in the context of sequence-based molecular markers such as nuclear ribosomal DNA internal transcribed spacer (ITS). Addressing this limitation is essential for a more accurate assessment of the breeding potential and adaptive capacity of this otherwise genetically constrained bioenergy crop.

This study was motivated by preliminary observations of M × g accessions obtained from different European sources, among which one accession exhibited distinct morphological features, including stem pigmentation and trichome development, not observed in the others. These differences raised the question of whether such phenotypic variation reflects underlying genetic divergence or is solely environmentally induced, and thus prompted a more systematic comparative analysis.

Accordingly, the present study aimed to evaluate morphological and genetic variation among M × g accessions originating from Croatia, Ukraine, Germany, and the Czech Republic. It was hypothesized that detectable genetic variation exists among these accessions and that such variation is associated with phenotypic differences relevant to biomass production and environmental adaptation.

## Materials and methods

### Experimental design

Four M × g accessions were included in this study, originating from Croatia (St. Helena deposit field near Zagreb), Ukraine (Institute of Bioenergy Crops and Sugar Beet, Kyiv), Germany (Handels & Vertriebsgenossenschaft cooperative, Cheb), and the Czech Republic (Research Institute for Landscape, Průhonice), hereafter referred to as HR, UA, DE and CZ. These accessions were selected from plant material collected during previous research activities and obtained from international collaborators and local sources. The selection was therefore based on availability rather than a predefined systematic sampling strategy. The included accessions represent plants propagated independently across different European regions and maintained under distinct environmental and agronomic conditions, providing a relevant basis for comparative analysis of potential intra-specific variation.

Two rhizomes were planted in pots filled with 4 L of compost (KS garden substrate) and 500 mL of sand at the bottom for drainage purposes on April 29, 2021 and cultivated for one vegetation season in the greenhouse. Each accession was prepared in six replicates. Prior to the planting process, the rhizomes were sorted to select only those of comparable dimensions. In the course of the experiment, the plants were watered with distilled water bi-weekly in colder months, while in summer, they were watered every three to four days.

### DNA isolation and sequencing

To assess genetic diversity, the Internal Transcribed Spacer (ITS) rDNA region was utilized as a classical molecular marker. Genomic DNA isolation was performed using three randomly selected plants from each accession. The 1 g of selected fresh healthy-looking leaf was used for the isolation. The genomic DNA isolation procedure employed a conventional CTAB technique as outlined by Doyle and Doyle [29]. A quantity of 100 ng of DNA was utilized as the template for the polymerase chain reaction to multiply regions of rDNA (ITS1 and ITS2). Due to indel described by Hodkinson [11], it was unable to sequence the entire ITS1–5.8 S - ITS2 region. The PCR had to be performed in two independent experiments. Primers used for ITS1 PCR were following: 18Sfor primer 5′-GCGCTACACTGATGTATTCAA CGA G-3′ and 5.8 S rev primer 5′-CCGTTGCCGAGAGTCGTTT-3′. Primers for amplification of ITS2 PCR were: 5.8Sfor 5′-GCATCGATGAAGAACGCAGC-3′ together with 26Srev primer with sequence 5′-CTTTTCCTCCGCTTATTGATATGC-3′. For both loci, amplification was carried out using the Combi PPP master mix (Top Bio, Czech Republic). The Polymerase Chain Reaction (PCR) was conducted using a GeneTouch thermal cycler (Bioer, China). The PCR protocol involved an initial denaturation step of 180 s at 94 °C, followed by 30 cycles of amplification consisting of denaturation at 94 °C for 30 s, annealing at 57 °C (for ITS1) or 56 °C (for ITS2) for 30 s, and extension at 72 °C for 45 s. The amplification was concluded with a final extension step at 72 °C for 10 min. The PCR product underwent analysis on 2% agarose gel and was subsequently purified through the utilization of the QIAquick PCR gel clean-up kit, manufactured by QIAGEN (Germany).

For sequencing, same primers as for PCR reactions were used: the 18Sfor primer for sequencing of ITS1 and the 26Srev primer for sequencing ITS2 region. The sequencing samples were prepared in accordance with the instructions provided by Eurofins Genomics, Germany. The data were subjected to analysis using BioEdit software version 7.2.

The obtained raw chromatograms were subjected to quality control by manual inspection of peak clarity; low-quality ends were trimmed to ensure high-confidence base calling. For the ITS2 region, sequencing was performed using the 26Srev primer. As this is a reverse primer, the resulting raw sequences represented the reverse complement of the target rDNA region. Consequently, these sequences were reverse-complemented using the built-in function in BioEdit to match the orientation of the reference sequences prior to alignment. The processed sequences were then aligned using the ClustalW algorithm implemented within the BioEdit software (version 7.2). For the identification of genetic variation, the aligned sequences were compared against reference sequences (GenBank accession numbers AJ426562 and AJ426563), which represent previously described ITS ribotypes of *M. × giganteus* [11]. Polymorphic sites (SNPs or indels) were identified by a comparative visual inspection of the aligned sequences against the reference ribotypes. This stepwise approach ensured that all identified variations were verified against both the sequencing chromatograms and the established reference framework.

**Fig. 1:**
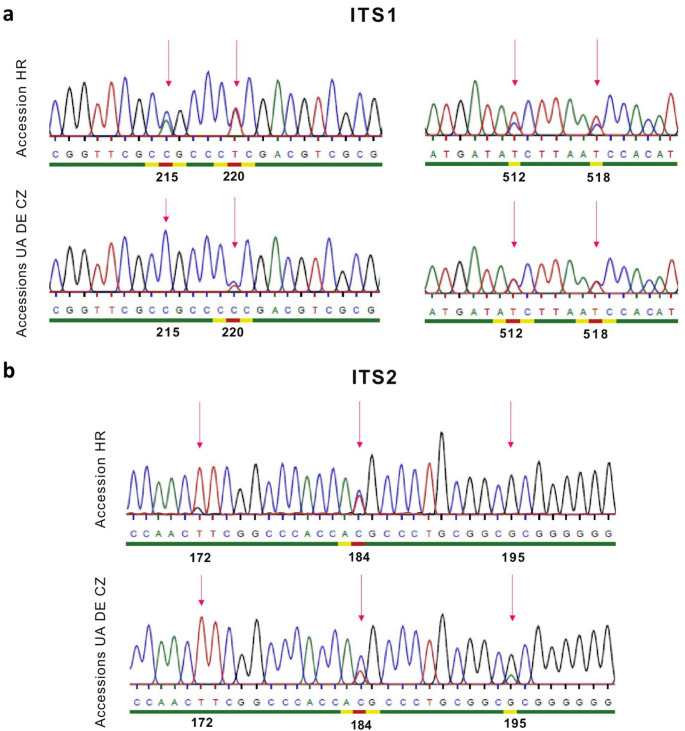
The differences in **(a)** ITS1 (215, 220, 512, and 518 bp) and **(b)** ITS2 (172, 184, and 195 bp) regions of accession HR and other tested accessions of *Miscanthus × giganteus*

Figure1; Table 1 include raw sequencing data without any manipulation (like reverse complement etc.).

### Biometric and morphological characteristics

Over a period of six months, from May to October, the number of sprouts and the height of plants in each pot were recorded between the 13th and the 16th day of each month. Upon completion of the experiment, the aboveground parts of the plants were harvested and weighed in terms of fresh (but naturally withered) biomass yield per pot.

The experiment also placed emphasis on morphological characteristics, including stem color, the existence of stem trichomes and marrow color, documented with the photographs.

During the experiment, a sample of three plants from each accession was randomly selected to investigate the number of leaf stomata on both the abaxial (lower) and adaxial (upper) sides of the leaves. The stomatal density was determined by counting the number of stomata per cm^2^, with three measurements taken for each leaf using inverted microscope INV100-FL (Bel Engineering, China) equipped with camera Eurokam 3.0 PLUS (Bel Engineering, Italy).

### Lignocellulose composition

The contents of lignin, cellulose and hemicelluloses in biomass samples were analyzed externally in AgroDigest s.r.o. laboratory (Czech Republic, Brno) by neutral detergent fiber (NDF), acid detergent fiber (ADF) and acid detergent lignin (ADL) analyses. The protocols followed the ČSN EN ISO 16,472 (2012) and ČSN EN ISO 13,906 (2011) standard procedures. Then, the cellulose content was calculated as ADF-ADL and hemicelluloses content as NDF-ADF. Four individual plants were sampled from each accession.

### Statistical analysis

The data of plant biometric parameters (biomass yield, plant height, and number of sprouts), stomatal density, and lignocellulose composition were statistically analyzed using OriginPro 2024 software (OriginLab Corporation, USA). For each parameter, differences among M × g accessions were evaluated using one-way analysis of variance (ANOVA). When significant differences were detected (*P* ≤ 0.05), means were compared using Tukey’s honestly significant difference (HSD) post-hoc test. Results are presented as mean ± standard deviation (SD), with the number of replicates indicated for each dataset. Graphical outputs were generated in OriginPro 2024. Statistically homogeneous groups identified by Tukey’s HSD test were indicated in the figures by different letters. Additionally, Pearson’s correlation analysis was performed to assess relationships among biometric parameters at harvest (biomass yield, plant height, and number of sprouts). The strength and direction of the relationships were expressed as Pearson’s correlation coefficient (r), and statistical significance was evaluated at *P* ≤ 0.05.

## Results

### Genetic analysis of *Miscanthus × giganteus* accessions

In the case of M × g, all accessions showed highly similar sequences, with variation detected primarily in accession HR. This necessitated a thorough analysis of all electrophoretograms. Double peaks consisting of two distinct letters can be observed at certain loci (Fig. 1). Sequencing of the ITS1 and ITS2 regions revealed that accession HR differed from the other accessions at multiple loci. While UA, DE, and CZ produced essentially identical sequences, HR displayed distinct polymorphisms in ITS1 at positions 215, 220, 512, and 518 bp (Table 1; Fig. 1a) and in positions 172, 184 and 195 bp in ITS2 (Table 1; Fig. 1b). For example, in ITS1 at position 215 bp, HR showed a mixture of C and A, whereas other accessions contained only cytosine. In the ITS2 region, position 172 bp possessed C and T in HR, whereas other accessions contained only thymine.

**Table 1:** The differences in the representation of DNA bases in each *Miscanthus × giganteus* accession within ITS1 and ITS2

Accession	Position of the sequence						
	ITS1				ITS2		
	215 bp	220 bp	512 bp	518 bp	172 bp	184 bp	195 bp
HR	C > A	T = C	T > C	T > C	T > G	T = C	G
UA	C	C > T	T = C	T = C	T	C > T	G > A
DE	C	C > T	T = C	T = C	T	C > T	G > A
CZ	C	C > T	T = C	T = C	T	C > T	G > A

* In silico* comparison of the obtained ITS sequences with reference sequences AJ426562 (ribotype A) and AJ426563 (ribotype G) from Hodkinson et al. [11] revealed several consistent differences among the analyzed accessions (see Supplementary file 1). In the ITS1 region, polymorphic sites were detected for example at positions 215, 220, and 518 bp. In the ITS2 region, at position 195 bp, accessions UA, DE, and CZ showed G/A heterozygosity, whereas accession HR displayed fixation of the G variant without detectable A signal. At position 184 bp, Hodkinson reports either T (AJ426562) or C (AJ426563), whereas chromatograms in this study consistently showed both nucleotides (C and T).

### Biometric parameters

**Fig. 2:**
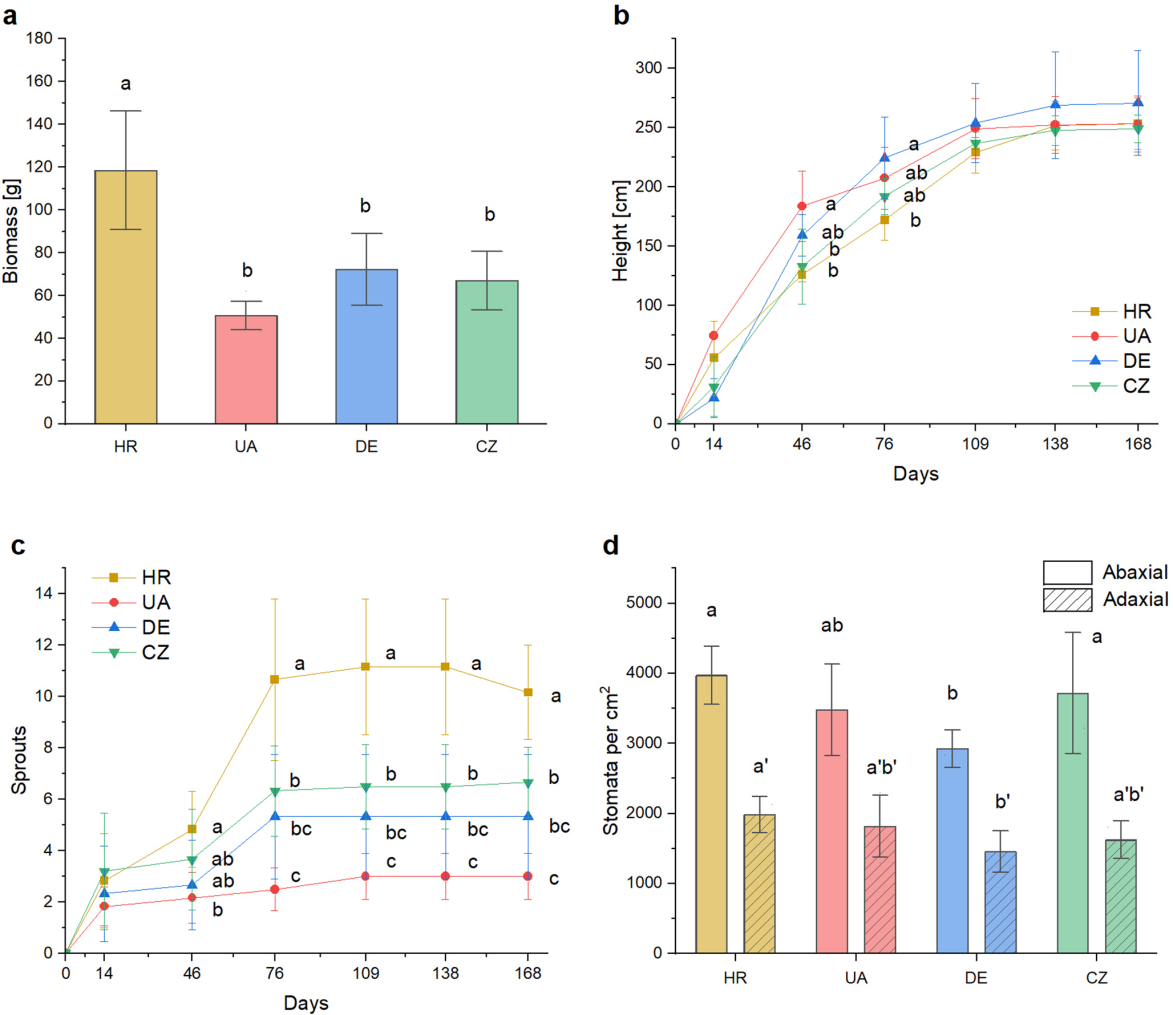
Biometric parameters of *Miscanthus × giganteus* accessions: **(a)** biomass yield per pot; **(b)** plant height; **(c)** number of sprouts and **(d)** stomatal density on abaxial and adaxial leaf surface; mean ± SD, different letters refer to significant differences between accessions at the time of measurement (ANOVA and Tukey HSD test, *n* = 6 (a, b, c) or 9 (d), *P* ≤ 0.05); the x-axis ticks at b, c show the days of parameters measurement

Notable variations between the M × g accessions were recorded in the biometric parameters including biomass (Fig. 2a), plant height (Fig. 2b) and sprout number (Fig. 2c) development during the vegetation season. In particular, the accession HR significantly outperformed the others in terms on biomass with yields approximately 1.6–2-fold higher than those of the other accessions. This increase was primarily associated with a significantly higher number of sprouts, which was 1.5-3-fold higher than in the other accessions and showed a strong positive correlation with biomass (*r* = 0.73, *P* < 0.001). In contrast, plant height was not a key determinant of biomass production, as it was comparable among all the accessions at the end of vegetation season, when biomass was harvested, and exhibited no significant correlation with biomass (*r* = 0.11, *P* = 0.60). Notably, despite its highest biomass yield, the HR accession exhibited shorter shoots during the main phase of the vegetation season (days 46–106). Conversely, the least productive UA accession showed the lowest sprouting capacity. Interestingly, UA plants were the tallest during the early growth stages (the first 46 days), when new sprouts were forming, suggesting a trade-off between sprouting intensity and shoot elongation.

### Morphological traits

**Fig. 3:**
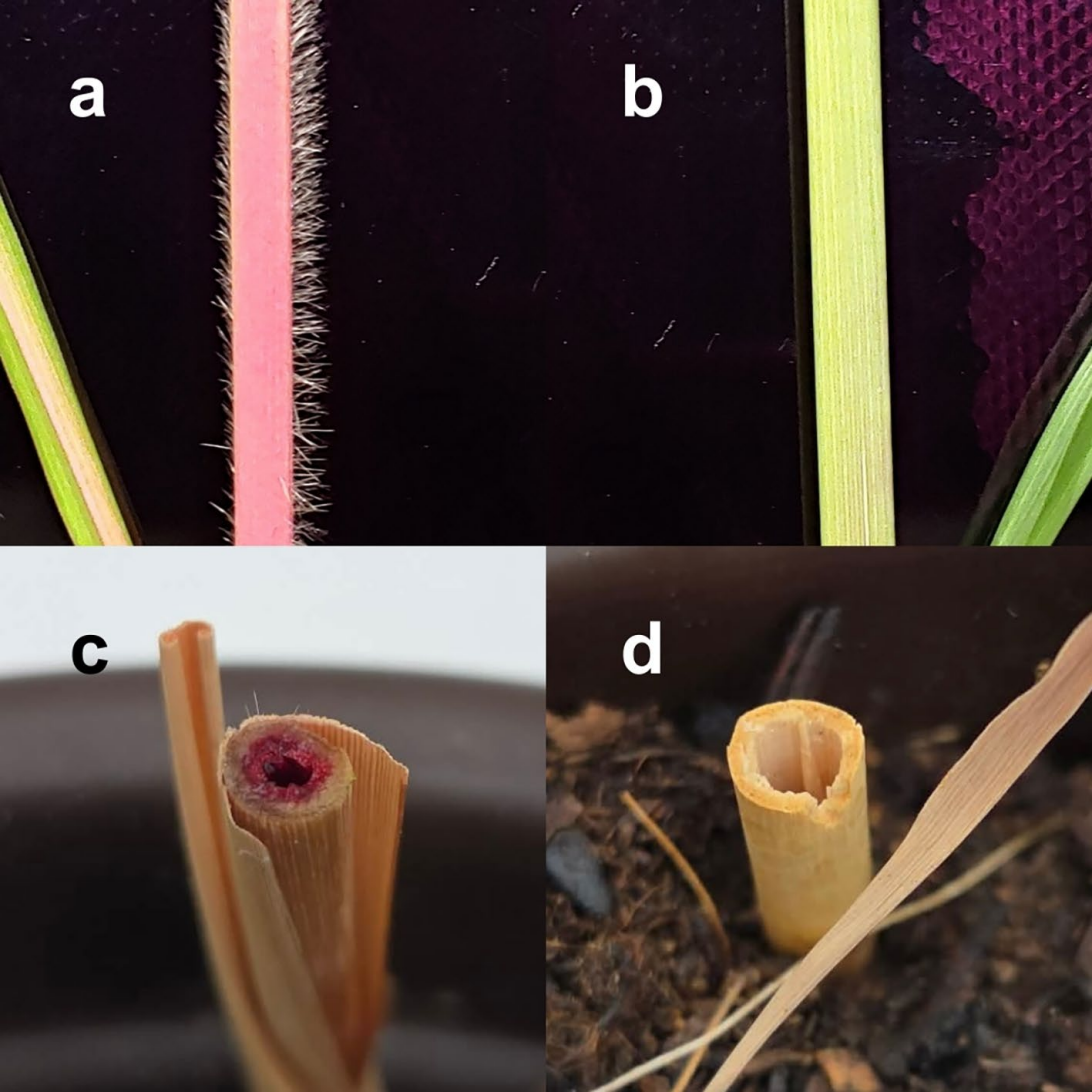
Morphological differences in stem color and presence of trichomes observed in **(a; c)** accession HR and **(b; d)** accession CZ

Following the biometric parameters, it was observed that there were also apparent morphological distinctions between HR and the other accessions (Fig. 3). The primary disparities referred to the stems. Accession HR had a dark purple hue and was characterized by the presence of trichomes throughout its entire length (Fig. 3a), a trait often associated with enhanced protection against abiotic stress and reduced water loss [30–32]. On the contrary, the stem of other accessions exhibited a green and unblemished appearance devoid of trichomes (Fig. 3b). Differences in stem marrow color were also observed. Accession HR exhibited a distinct characteristic whereby its marrow displayed a dark pink to a purple hue (Fig. 3c), which is commonly linked to anthocyanin accumulation and photoprotective responses [30], in contrast to other variants that feature light stem marrows represented by accession CZ in Fig. 3d.

The highest mean stomatal density on both, abaxial and adaxial, leaf surfaces (Fig. 2d) was observed in accession HR. However, it did not differ significantly from that of accessions UA and CZ. In contrast, the accession DE exhibited a significantly lower stomatal density, especially on the abaxial leaf surface.

### Lignocellulose composition

Despite significant differences of accession HR observed in biometric and morphological parameters, the contents of lignocellulose components in biomass samples (Table 2) were comparable among all the accessions. Although, HR had the lowest mean lignin/holocellulose ratio, with value 0.09 ± 0.02 compared to 0.12 ± 0.01 in the other accessions.

**Table 2:** Contents of lignocellulose components in *Miscanthus × giganteus* biomass; mean ± SD, no significant difference was determined (ANOVA, *n* = 4, *P* ≤ 0.05)

Accession	Lignin [%]	Cellulose [%]	Hemicelluloses [%]
HR	7.0 ± 1.3	44.9 ± 1.4	29.9 ± 1.9
UA	8.8 ± 0.9	43.9 ± 1.1	28.8 ± 0.8
DE	8.1 ± 1.0	44.8 ± 2.2	28.5 ± 0.9
CZ	8.8 ± 0.3	45.2 ± 0.9	27.0 ± 0.8

## Discussion

### Interpretation of genetic variability

The presence of double peaks and distinct polymorphisms is characteristic of polyploid organisms, as their genetic makeup comprises two or more distinct genomes. Given M × g’s triploid origin, it is not unexpected. However, the pattern of these polymorphisms differed among the analyzed accessions. Sequencing of the ITS1 and ITS2 regions revealed variation among the individual accessions, with the most pronounced differences observed in accession HR. Hodkinson et al. [11, 33] have previously identified comparable SNPs in M × g within the ITS regions, although those studies focused on broader intergenic variation.

The *in silico* comparison with the reference sequences AJ426562 (ribotype A) and AJ426563 (ribotype G) from Hodkinson’s study [11] further supports the presence of structured variation within the ITS region of the analyzed material. Unfortunately, due to use of different primers for PCR, our and Hodkinson’s sequences overlap only partially.

In general, UA, DE, and CZ accessions exhibited patterns consistent with heterozygosity between parental ribotypes, whereas accession HR showed a reduced level of ITS polymorphism, suggesting a shift toward fixation of a single ribotype signal. This indicates that ribosomal DNA variation may not be fully homogenized even within clonally propagated triploid material.

According to Mitros et al. [25], divergence between *M. sinensis* and *M. sacchariflorius* occurred approximately 1.65 million years ago. Prior research has indicated that there is significant variation in the genome size of *Miscanthus* species, including among various accessions of M × g [10, 22, 34]. M × g, known as a triploid hybrid, has the ability to assume diploid, triploid, or allotetraploid forms, contingent upon its chromosomal count. It is plausible that various ploidy levels may manifest within this species [11, 25, 35]. The observed loss of polymorphism at position 195 bp in the HR accession is particularly significant. It suggests that even within a sterile triploid clone, the ribosomal DNA is not static. This fixation of a single ribotype (G) could be a result of concerted evolution, a process where one sequence variant replaces another through unequal crossing-over or gene conversion, often seen in polyploids [36]. The observed molecular divergence in the HR accession coincides with its distinct morphological profile (e.g. slightly higher stomatal density). This pattern may reflect intra-genomic homogenization processes or lineage-specific variation. Alternatively, it could be associated with somatic variation accumulated during clonal propagation. The alignment of molecular divergence with distinct morphological traits in the HR accession suggests a functional link that warrants further attention. The observed fixation of a single ITS ribotype (G) and the simultaneous increase in stomatal density and tiller number could indicate that the genomic reorganization, likely driven by concerted evolution, is not limited to the rDNA loci. Instead, these changes may be proxy indicators of broader genomic reshuffling that has favored specific physiological adaptations, such as enhanced gas exchange capacity and increased biomass productivity, potentially providing a selective advantage in the warmer climatic conditions of Croatia.

Furthermore, the distinctiveness of the HR accession might not solely arise from a different lineage but could be explained by the segregation of alleles during the initial hybridization event. Given that both parental species, particularly *M. sacchariflorus*, often exhibit hybrid origins and high levels of heterozygosity [11, 35], the resulting M × g offspring can harbor significant genomic variation even if derived from the same parental cross. The HR accession may thus represent a specific transgressive segregant that has captured a unique combination of alleles, leading to its higher growth performance.

### Analysis of growth and performance

The observed biometric pattern aligns with previous findings by Al Souki et al. [37], where three different cultivars originating in England, USA and Austria, were similarly tall after two growing seasons, but one of them had significantly higher number of sprouts. It is known, that M × g biometric parameters can be highly affected by soil quality, nutrients availability and contamination [38, 39]. However, in the current experiment, all plants grew in the same soil providing sufficient amount of nutrients and lacking any contamination, suggesting that genetic factors may contribute to the observed variability in accession HR. Comparative studies of *M. sinensis*, *M. sacchariflorus*, and related *Miscanthus* species indicate that the association between plant height and sprout number is species-dependent [6]. Arnoult and Brancourt-Hulmel [17] postulated that stem number in certain *M. sinensis* cultivars was unexpectedly high. It can be speculated that similar cultivar of *M. sinensis* was one of the ancestors of accession HR. This advantage may be genetically inherited; however, it can also be influenced by environmental factors.

The identification of distinct ITS polymorphisms in the HR accession suggests that the perceived genetic bottleneck in European M × g plantations may not be absolute. This may indicate previously unrecognized genetic variation, potentially arising from distinct initial hybridization events or somatic mutations stabilized during long-term clonal propagation.

Furthermore, although the HR accession represents the most striking case of divergence, subtle phenotypic variations were also observed among the UA, DE, and CZ accessions, even if these did not reach statistical significance in most biometric parameters. It remains plausible that these minor morphological and physiological differences are underpinned by smaller genetic variations not captured in our study. As our molecular analysis was specifically targeted at the ITS1 and ITS2 regions of the nuclear rDNA, which are relatively conserved, further investigation using higher-resolution genomic tools, such as Genotyping-by-Sequencing (GBS) or SSR markers, would be necessary to fully map the finer scale of genetic heterogeneity among these accessions.

### Morphological and physiological variation and environmental adaptation

Stomata play a crucial role in the survival of land plants because their function is essential for gas exchange. Previous research has demonstrated that the number of stomata in M × g leaves increases when cultivated in soil with low nutritional quality [40], but variability between different accessions was not examined. The higher stomatal density may provide these accessions an environmental advantage, especially while facing higher temperatures and drought. The highest stomatal density in the HR suggests a potential higher capacity for gas exchange, which in C4 grasses like *Miscanthus* often correlates with enhanced photosynthetic rates under optimal light and temperature conditions [41, 42]. This physiological trait may be associated with the observed differences in biomass production, representing a significant deviation from the standard M × g clones. It could be speculated that this pattern was related to the origin of HR accession in Croatia, which has a comparatively warmer climate than the other regions of origin. Thus, such differences may reflect partial adaptation of this accession to its local environmental conditions.

This theory can be supported also by other morphological traits: the distinctive dark purple pigmentation observed in the stems and marrow of the HR accession is likely attributed to the accumulation of anthocyanins. These flavonoid pigments are widely recognized for their multifaceted roles in plant physiological responses, particularly as antioxidants and photoprotectants. Anthocyanin deposition in vegetative tissues is frequently induced as a response to environmental stressors, such as high light intensity or fluctuating temperatures, and serves to mitigate oxidative damage [30, 42]. In the context of the HR accession, this trait, alongside its unique morphological features such as the presence of trichomes, may indicate a potentially distinct physiological response pattern. Such physiological characteristics may be associated with improved performance under the given conditions and with the higher biomass yield observed in this accession.

### Lignocellulose and biomass composition

Modulation of lignocellulose content in M × g stems was observed after application of various types of stress [32], however, the response of different accessions grown under the similar conditions is unknown. The variable contents of lignin, cellulose and hemicelluloses were also documented between different *Miscanthus* species [17]. In contrast, no statistically significant differences in terms of lignocellulose composition were observed among the M × g accessions in the present study.

The lack of significant differences does not necessarily compromise biomass quality. This is a crucial finding for the bioenergy industry, as it suggests that high-yielding genotypes like HR can be integrated into existing processing pipelines without requiring technical adjustments.

## Conclusions

This study compared four accessions of *Miscanthus × giganteus* (M × g) originating from Croatia, Ukraine, Germany, and the Czech Republic, revealing that despite the generally accepted assumption of genetic uniformity, measurable differences exist among European clonal lines. The Croatian accession was consistently distinguished not only by higher biomass yield and increased sprouting capacity, but also by distinct morphological traits and unique ITS sequence polymorphisms.

These results indicate that intra-clonal variation within M × g may be greater than previously assumed and could have functional consequences for biomass productivity and plant performance. Importantly, the detected genetic differences suggest that even within a predominantly sterile, clonally propagated crop, divergent lineages may exist within European clonal material, potentially arising from historical propagation events or genomic variation processes.

Future research should focus on high-resolution genomic approaches to clarify the origin and stability of the observed polymorphisms. In particular, cytogenetic methods such as Fluorescent In Situ Hybridisation (FISH) should be applied to determine the chromosomal organization and localization of rDNA loci in divergent accessions. In addition, genome-wide analyses (e.g. GBS or whole-genome sequencing) across a broader panel of European M × g clones are recommended to assess the extent of hidden genetic diversity and its relationship to agronomic traits.

Overall, these findings suggests that the extent of genetic and phenotypic uniformity in M × g may be more complex than previously assumed and should be considered particularly in the context of breeding and long-term bioenergy crop improvement.

## Supplementary Information

Below is the link to the electronic supplementary material.

Supplementary Material 1

## Data Availability

All data will be available upon reasonable request.
