# Supplementary material for: Genetic diversity within Miscanthus × giganteus: Evidence from morphological traits and ITS rDNA in European accessions
Source: Mol Biol Rep. 2026 Jul 15;53(1):1174. doi: 10.1007/s11033-026-12301-z (PMC13372840; doi:10.1007/s11033-026-12301-z)

Comparison of ITS2 sequences

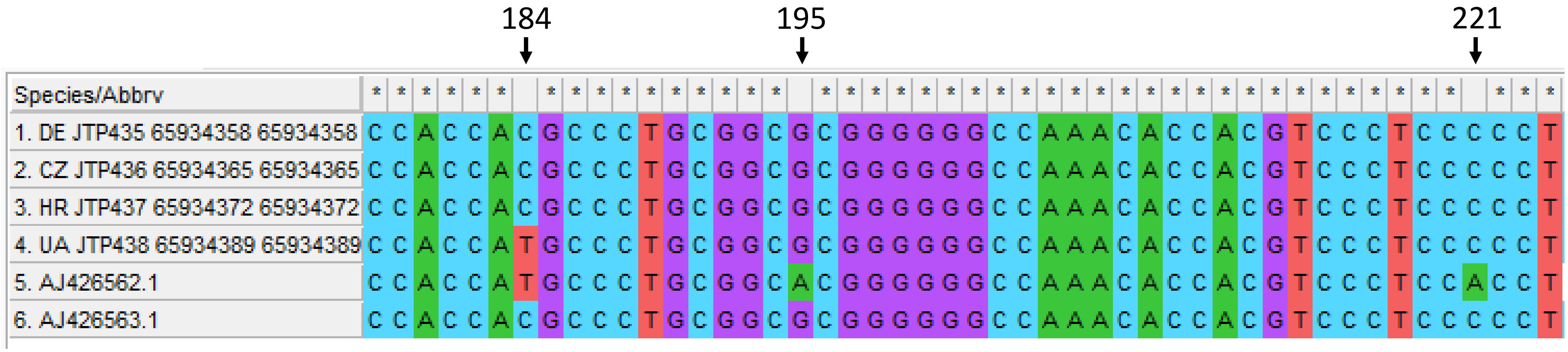

Supplement: Supplementary file 1 — Supplementary Material 1 [file 11033_2026_12301_MOESM1_ESM.pdf]
